# Using a continuum of hybrid effectiveness-implementation studies to put research-tested colorectal screening interventions into practice

**DOI:** 10.1186/s13012-019-0903-5

**Published:** 2019-05-29

**Authors:** Beverly B. Green, Gloria D. Coronado, Malaika Schwartz, Jen Coury, Laura-Mae Baldwin

**Affiliations:** 1 Kaiser Permanente Washington Health Research Institute, Kaiser Permanente Washington, Seattle, Washington USA; 2 Kaiser Permanente Center for Health Research, Kaiser Permanente Northwest, Portland, Oregon USA; 3 Department of Family Medicine, University of Washington School of Medicine, Seattle, Washington USA; 4 CareOregon, Portland, Oregon USA

**Keywords:** Implementation trial, Adaptation, Adoption, Scale-up, Comparative effectiveness research/methods, Colorectal cancer, Screening

## Abstract

**Background:**

Few previous studies have applied the hybrid effectiveness-implementation design framework to illustrate the way in which an intervention was progressively implemented and evaluated across multiple studies in diverse settings.

**Methods:**

We describe the design components and methodologies of three studies that sought to improve rates of colorectal cancer (CRC) screening using mailed outreach, and apply domains put forth by Curran et al.: research aims, research questions, comparison conditions, sample, evaluation methods, measures, and potential challenges.

The Hybrid 1 study (emphasis on effectiveness) was a patient-level randomized trial of a mailed fecal test and stepped phone-outreach intervention program delivered in an integrated healthcare system (21 clinics, 4673 patients). The primary outcome was effectiveness (CRC screening uptake). Implementation outcomes included cost-effectiveness and acceptability.

The Hybrid 2 study (shared emphasis on effectiveness and implementation) was a pragmatic cluster-randomized trial of mailed fecal immunochemical test (FIT) outreach implemented at safety net clinics (26 clinics, 41,000 patients). The intervention used electronic health record tools (adapted from Hybrid 1) and clinic personnel to deliver the intervention. Outcomes included effectiveness (FIT completion) and implementation (FIT kits delivered, clinic barriers and facilitators, cost-effectiveness).

Hybrid 3 study (emphasis on implementation) is a demonstration project being conducted by two Medicaid/Medicare insurance plans (2 states, 12,000 patients) comparing two strategies for implementing mailed FIT programs that addressed Hybrid 2 implementation barriers. Outcomes include implementation (activities delivered, barriers) and effectiveness (FIT completion).

**Results:**

The effectiveness-implementation typology successfully identified a number of distinguishing features between the three studies. Two additional features, program design and program delivery, varied across our studies, and we propose adding them to the current typology. Program design and program delivery reflect the process by which and by whom a program is designed and delivered (e.g., research staff vs. clinic/health plan staff).

**Conclusions:**

We describe three studies that demonstrate the hybrid effectiveness to implementation continuum and make recommendations for expanding the hybrid typology to include new descriptive features. Additional comparisons of Hybrid 1, 2, and 3 studies may help confirm whether our hybrid typology refinements are generalizable markers of the pipeline from research to practice.

Contributions to the literature

- We describe three studies that demonstrate the hybrid effectiveness to implementation continuum framework and recommend adding two new domains: program design and program delivery.
- Hybrid 1 study focused on the effectiveness of direct-mailing of fecal tests to increase colorectal cancer screening. Hybrid 2 adapted the program to community settings, evaluating implementation and effectiveness. Hybrid 3 is evaluating strategies designed to address Hybrid 2 study barriers.
- Program design and delivery became increasingly pragmatic, adaptable, and informed by contexts: researcher-led in Hybrid 1; collaborative in Hybrid 2; and organization-led in Hybrid 3.
- Consideration of these two new hybrid domains could speed the transition from research to practice.

## Background

Strong evidence indicates that colorectal cancer (CRC) screening decreases CRC morbidity and mortality. However, only 62% of age-eligible adults are current for screening, with lower rates among low-income (47%), uninsured (25%), and Hispanic populations (47%) [1]. Effective strategies for improving CRC screening uptake and decreasing disparities are needed, as is research on whether the most effective strategies can be adapted, implemented, and maintained in diverse settings.

Much has been written about difficulties in translating evidence-based practices and research-tested programs into routine care. From the time evidence is generated to the time it becomes part of everyday practice is estimated to take longer than 17 years [2]. Reasons for this delay include that (1) resources used to implement an effective program in a research setting may not be available in community settings, (2) the original intervention was tested in a unique setting (such as an academic center) and under optimal conditions that are not generalizable to other settings, and (3) the research included patients that are not representative of the general population or did not test the program with disadvantaged groups (such as those with language barriers or who lack health insurance).

We describe the design components and methodologies of three studies that sought to move a research-tested program into practice. In our description, we apply the hybrid effectiveness-implementation design models as described by Curran et al. [3]. The hybrid model provides a framework for designing studies that have dual purposes of testing intervention effectiveness and implementation strategies to accelerate knowledge creation and increase the relevance of research [3]. Hybrid studies provide a pathway for rapidly moving knowledge from research to implementation. Hybrid 1 studies generally focus on the effectiveness of a clinical intervention in a setting where it will be implemented, while secondarily exploring some aspects of implementation (e.g., fidelity of intervention delivery, costs). The main emphasis in this type of study is the following: *Is the clinical intervention effective*? Hybrid 2 studies focus on both implementation and effectiveness outcomes, and generally ask the following: *Does the implementation method facilitate implementation of the intervention and is it still effective?* Hybrid 3 studies focus on comparing different implementation strategies for scaling up and maintaining an intervention. We present here three pragmatic studies designed along this continuum, with each sequentially informing the next research questions and study design [4]. We recommend ways for augmenting the hybrid effectiveness-implementation design framework to include two additional categories: program design and program delivery.

## Methods

We first described key characteristics of three studies using the hybrid effectiveness-implementation design continuum, including the design, setting, data sources, participants, intervention/program, and outcomes. We then used Curran et al.’s published domains (Table 1) to characterize these Hybrid 1, 2, and 3 studies. These domains include research aims, research questions, units of randomization, comparison conditions, sample, evaluation methods, measures, and potential challenges. Finally, we compared the characteristics of the three hybrid studies along these domains and considered how our studies fit with the hybrid typology. This analysis led us to identify two additional domains, program design and program delivery, that distinguished our three studies along the Hybrid 1 through 3 continuum, as described below.

**Table 1:** Hybrid design characteristics and key challenges as described by Curran [3] and abridged by the authors

Study characteristic	Hybrid trial Type 1	Hybrid trial Type 2	Hybrid trial Type 3
Research aims	Primary aim: determine effectiveness of a clinical intervention Secondary aim: better understand context for implementation	Co-primary aim: determine effectiveness of a clinical intervention Co-primary aim: determine feasibility and potential utility of an implementation intervention/strategy	Primary aim: determine utility of an implementation intervention/strategy Secondary aim: assess clinical outcomes associated with implementation trial
Research questions (examples)	Primary question: will a clinical treatment work in this setting among these patients? Secondary question: what are potential barriers/facilitators to a treatment’s widespread implementation?	Co-primary question: will a clinical treatment work in this setting/these patients? Co-primary question: does the implementation method show promise (either alone or in comparison with another method) in facilitating implementation of a clinical treatment?	Primary question: which method works better in facilitating implementation of a clinical treatment? Secondary question: are clinical outcomes acceptable?
Units of randomization	Patient, clinical unit	Clinical effectiveness: see type I Implementation: see type III, although may be nonrandomized, for example, case study	Provider, clinical unit, facility, system
Comparison conditions	Placebo, treatment as usual, competing treatment	Clinical effectiveness: see type I Implementation: see type III, although may be nonrandomized, for example, case study	Provider, clinical unit, facility, system: implementation as usual, competing implementation strategy
Sampling frames	Patient: limited restrictions, but some inclusion/exclusion criteria Provider, clinical unit, facility, system: choose subsample from relevant participants	Patient: limited restrictions, but some inclusion/exclusion criteria. Providers/clinics/facility/systems; consider “optimal” cases	Provider/clinic/facility/system: either “optimal” cases or a more heterogeneous group Secondary: all or selected patients included in study locations
Program design	Program is largely designed by the research team, but can be informed by program sites	Program is designed collaboratively by researchers and program site and adapted to setting and populations.	Implementation strategies may be designed by the research team, collaboratively by the research team and program site, or by the program site with the research team as consultants and or evaluators.
Program delivery	Program is largely delivered by the research team with attention to fidelity	Delivered by the program site, with support from the research team. Fidelity may be variable, with reasons for variability explored	Delivered by the program site with variable support from the research team. Fidelity may be variable, with reasons for variability explored
Evaluation methods	Primary aim: quantitative, summative Secondary aim: mixed methods, qualitative, process-oriented	Clinical effectiveness aim: quantitative, summative Implementation aim: mixed method; quantitative, qualitative; formative and summative	Primary aim: mixed method, quantitative, qualitative, formative, and summative Secondary aim: quantitative, summative
Measures	Primary aim: patient symptoms and functioning, possibly cost Secondary aim: feasibility and acceptability of implementing clinical treatment, sustainability potential, barriers and facilitators to implementation	Clinical effectiveness aim: patient symptoms and functioning, possibly cost-effectiveness Implementation aim: adoption of clinical treatment and fidelity to it, as well as related factors	Primary aim: adoption of clinical treatment and fidelity to it, as well as related factors Secondary aim: patient symptoms, functioning, services use
Potential design challenges	Generating “buy in” among clinical researchers for implementation aims. Insuring appropriate expertise on study team to conduct rigorous Secondary aim. These studies will likely require more research expertise and personnel, and larger budgets, than non-Hybrids	Generating “buy in” among implementation researchers for clinical intervention aims. These studies will require more research expertise and personnel, as well as larger budgets, than non-Hybrids. Insuring appropriate expertise on study team to rigorously conduct both aims “Creep” of clinical treatment away from fidelity needed for optimal effectiveness. IRB complexities with multiple types of participants	Primary data collection with patients in large, multisite implementation trials can be unfeasible, and studies might need to rely on subsamples of patients, medical record review, and/or administrative data. Patient outcomes data will not be as extensive as in traditional effectiveness trials or even other Hybrid type and might be insufficient to answer some questions. “Creep” of clinical treatment away from fidelity needed for optimal effectiveness. Institution review board complexities with multiple types of participants

*Program design* considers the initial design of the program or intervention, and further adaptations for varied settings and populations. In the effectiveness to implementation continuum of our studies, we identified varying levels of collaboration between the program sites and researchers. This continuum went from researchers determining the design, to a shared process, to researchers mainly serving as consultants and/or evaluators. *Program delivery* includes the specification of the who, what, where, when, and how the program is delivered in each setting and is in part informed by the work of Proctor and others in specifying implementation components [5, 6]. For example, the who in Proctor is called the actor—or the individual implementing the strategy, which might be the research team or individuals within the setting with or without research team support or training. The what, when, and how the strategy is delivered may also vary across studies, with some studies focusing mainly on effectiveness and consistent delivery of interventions, and other studies focusing on real-world implementation of various program delivery strategies.

### Description of the Hybrid 1, 2, and 3 study examples

Multiple prior publications provide detailed descriptions of the Hybrid 1 and 2 studies presented here; the Hybrid 3 study is on-going [7–9]. A brief description of each study is provided below. We also describe the two new domains, program design and program implementation as these were distinctly different between the three studies.

The Hybrid 1 study (Systems of Support to Increase Colorectal Cancer Screening, SOS, R01 CA121125) was a 4-arm patient-level randomized trial (4675 patients). It was conducted from 2008 to 2011 in an integrated health care system that provides both health insurance and health care (primary and specialty care, including colonoscopies) [7, 10]. The research staff used automated data (electronic health record [EHR] data, laboratory data, and procedural CPT codes) to identify age-eligible patients not current for CRC screening and evaluate outcomes. The intervention was embedded in the health care system and used an EHR-linked registry to deliver a centralized program to encourage CRC screening uptake. Usual care (Arm 1) received annual birthday reminders of overdue screening tests including CRC screening. In addition to usual care, participants randomized to active interventions received stepped intensity interventions: information on CRC screening choices, mailed fecal kits, postage-paid return envelopes, and a mailed reminder if the kit was not returned (Arm 2); mailings plus brief telephone assistance if screening was not completed after the mailings (Arm 3); mailings plus brief assistance plus nurse navigation for those still not completing screening (Arm 4). The research team managed the database with a vendor service mailing the intervention components; clinic medical assistants and nurses worked from their regular clinic but had protected time (the study paid for about 4–8 h a week of their time) to provide telephone assistance and navigation to patients across the organization [7]. The primary outcome was CRC screening adherence over 2 years in each of the progressive intensity arms compared to usual care (Arm 1). Secondary implementation outcomes included the reach of the intervention, cost-effectiveness from the health plan’s perspective, qualitative assessments from the patients’ perspective of how the intervention could be improved, and the intervention’s fit within the organization’s other quality improvement efforts to increase CRC screening [10–14]. The mailed program alone doubled CRC screening uptake, with incremental increases in the stepped intensity groups [10]. Mailed interventions and calls from medical assistants or nurses from other clinics were acceptable to patients if the medical assistant or nurse had access to their EHR record and could communicate with the patient’s physician [13].

The Hybrid 2 study (Strategies and Opportunities to STOP Colon Cancer in Priority Populations, UH3 AT007782), was a pragmatic cluster-randomized trial conducted from 2014 to 2016 at 26 safety net clinics with over 40,000 patients in the first year who were age-eligible and overdue for CRC screening [8, 15]. EHR and laboratory data were used to identify eligible patients and to evaluate effectiveness outcomes. The intervention was adapted in part from the Hybrid 1 SOS study, in that it used EHR data to identify individuals overdue for CRC screening and a registry to automatically generate mailings. However, the research team, EHR vendor, and clinic staff collaboratively designed and implemented the program for use in community clinic settings and by diverse patients. Adaptations included translating patient letters into Spanish and other languages, using wordless fecal immunochemical test (FIT) pictographic instructions [16], and embedding the registry into the EHR (Reporting Workbench) to generate lists of patients overdue for CRC screening and to create mailings. An advisory committee made up of organizational and clinic program leaders, CRC screening experts, and patients reviewed all materials and suggested changes or enhancements. Clinic staff generated lists and completed mailings, with the research team only providing initial training, monthly clinic staff “champion” meetings to augment training and troubleshoot issues with the EHR-embedded tool, and facilitation of Plan-Do-Study-Act (PDSA) cycles to help the clinics improve program implementation [17]. The primary outcomes were FIT completion (effectiveness) and implementation outcomes based on the Reach, Effectiveness, Adoption, Implementation, and Maintenance (RE-AIM) framework (e.g., percent of clinics participating, percent of letters mailed by each clinic, percent of clinics continuing the program) and the Consolidated Framework for Implementation Research (CFIR) to assess contextual factors related to implementation (e.g. barriers and facilitators). Additional implementation outcomes included program cost-effectiveness. The intervention overall led to significantly higher rates of FIT completion compared to usual care clinics [15], but the net difference was modest (net increase 3.4%), mainly because implementation varied greatly across intervention clinics, with the health center-level percent of eligible intervention participants mailed a FIT ranging from 6.5 to 68.2% [15]. Among the patients who were mailed FITs, completion rates were close to the Hybrid 1 SOS study, 21% [15]. Mixed methods summative analyses of barriers and facilitators to implementation are underway, but qualitative rapid cycle assessments during the study suggested that contributing factors were delays in start-up, personnel changes and staffing shortages, and challenges with the technologies and lab ordering processes [18].

The Hybrid 3 study began in 2015 and is an on-going evaluation of two different mailed FIT programs being implemented by two Medicaid/Medicare insurance plans in 2 states (BeneFIT, UDP-14-0012) [9], with age-eligible health plan members identified and outcomes evaluated using claims data (12,000 patients in year 1). The investigators initiated the relationship with the health plans with the goal of addressing implementation barriers experienced in the Hybrid 2 study, specifically that clinics often had insufficient staff or resources to consistently implement the program. One program is working collaboratively with clinics to integrate it into their usual workflow (e.g., a vendor mails the same FIT kit type used in clinical care, and completed tests are mailed back to the clinics, where staff place test orders and deliver follow-up care). The other is a centralized program whose vendor mails the FITs, which are sent to a centralized laboratory for processing, then sends the results to the providers and directly contacts patients with abnormal results. Mixed methods outcomes include percent of age-eligible patients receiving mailings, acceptability of and satisfaction with the program by the health plans, implementation barriers and facilitators, effectiveness (FIT and colonoscopy completion rates), and program costs. Contextual factors related to implementation and maintenance of the programs are assessed using the CFIR framework.

## Results

### Hybrid design model characteristics of the 3 study examples

Table 2 compares each of the three reported hybrid studies across key design characteristics and shows differences with progression from Hybrid 1 to Hybrid 3 designs. SOS, the Type 1 hybrid study, was a person-level randomized controlled trial that invited almost all age-eligible individuals’ overdue for CRC screening and had few exclusions (CRC, inflammatory bowel disease, life-threatening health conditions). Outcomes were primarily effectiveness, but mixed methods were used to understand screening barriers and facilitators and to further refine study interventions in subsequent study years [13]. Cost-effectiveness evaluations were performed from a health care organization perspective to assist with decisions about adoption of the overall program and its specific components (such as the incremental cost-effectiveness of adding phone assistance and navigation) [12]. Challenges included the requirement of verbal consent by the institutional review board, with only 38% of those invited agreeing to participate [19]. Non-white and Hispanic individuals, and individuals with lower levels of education, were less likely to participate than non-Hispanic white individuals and patients with higher levels of education.

**Table 2:** Examples of Hybrid 1, 2, and 3 studies with recommendations for additional categories (shaded)

Study characteristics	Hybrid study 1	Hybrid study 2	Hybrid study 3
Name	Systems of Support to Increase Colorectal Cancer Screening and Follow-up (SOS)	Strategies and Opportunities to STOP Colon Cancer in Priority Populations (STOP)	Benefit for Increasing Colorectal Cancer Screening in Priority Populations (BeneFIT)
Research aims	To compare the effectiveness of an EHR-linked automated mailed and stepped intensity CRC screening program to usual care	To compare the Adoption, Reach, Implementation, and Maintenance of a clinic-based EHR-embedded mailed program to usual care	To evaluate implementation of mailed FIT programs by two health insurance plans
Research questions	Primary question: Will an automated mailed CRC screening program increase screening uptake? Implementation questions: What is the incremental cost-effectiveness of the stepped intensity program? Is the program acceptable to patients and how could it be improved?	Primary question: Compared to usual care in safety net clinics can a mailed fecal testing program be adapted and implemented in safety net clinics and will it increase CRC screening uptake in vulnerable eligible adults and compared to usual care in safety net? Co-primary question: What contextual factors influence adoption, reach, implementation, and maintenance of the program?	Primary question: Can different implementation strategies be used to increase the adoption, reach, effectiveness, and maintenance of a mailed FIT program? What do the programs cost? What contextual factors (inner and outer setting) influence program implementation? Secondary question: What is the FIT return rate among eligible health plan enrollees?
Unit of randomization (or comparison)	Patient (patient-level randomized trial)	Clinic (clinic-level randomized trial)	Naturalistic experiment (no randomization) with comparative outcomes
Comparison condition	Usual care	Usual care	Two different implementation models, no usual care comparator
Sampling frames	Patients age-eligible not current for CRC screening, limited number of exclusions	Clinics with sufficient numbers of age-eligible patients not current for CRC screening, almost no exclusion	Two Medicaid/Medicare health plans and their enrollees within two states - age eligible and not current for CRC, almost no exclusions
Program design	Research team designed	Designed through collaboration between the EHR vendor, clinics, and research team	Health plan designed, with the researchers serving as consultants
Program delivery	Research team delivered the mailings. The research team closely supervised clinical staff and the study paid for their time. Program fidelity was monitored; variation minimized.	Delivered by clinics with training assistance and implementation facilitation (plan, do, see, act cycle) by the research team. Program delivery fidelity varied	Delivered by the health plans with the research team providing consultative assistance. The health plans managed program components and program fidelity
Evaluation method	Quantitative and summative	Mixed methods: quantitative and qualitative, formative and summative	Mixed methods: quantitative and qualitative, formative and summative
Measures	Comparative colorectal cancer screening rates and cost-effectiveness	FIT screening rates overall and variation by clinic implementation and patient characteristics, RE-AIM measures, variation in costs by clinic, qualitative assessments of factors related to clinic success and challenges	RE-AIM measures comparing the two implementation models. Costs of implementation. Comparisons of FIT test return rates across the two implementation models
Potential design challenges	Verbal consent required, leading to decreased Reach and external validity	Variation in implementation fidelity. IRB was not a challenge: single institutional review board, waiver of consent	Quasi-experimental design might lead to bias. Implementation and effectiveness outcomes were not directly comparable between the two health plans (no randomization, no control comparison)

*Abbreviations*: *CRC* colorectal cancer screening, *FIT* fecal immunochemical test, *RE-AIM* Reach, Effectiveness, Adoption, Implementation, Maintenance

STOP CRC, the Hybrid 2 study, sought to adapt and implement the SOS program within community health center clinics that care for populations with the lowest CRC screening rates. STOP CRC had almost no exclusions and consent was waived, making it possible to reach nearly 100% of the population that needed the intervention. This study measured both overall effectiveness of the program compared to control clinics, (i.e., FIT uptake) and variation in effectiveness across health centers [18]. Implementation was measured as the proportion of eligible patients who were mailed a FIT (quantitative) at each health center and their barriers and facilitators to implementation (qualitative) [16] and maintenance (RE-AIM). Early in the study, it became obvious that implementation fidelity was the primary challenge, with some clinics delaying start-up until almost the end of the participant accrual interval. These delays and implementation variability led to reductions in program effectiveness.

In BeneFIT, the Hybrid 3 study, we are evaluating mailed FIT programs designed to decrease the burden of implementation delivery on clinics by enlisting Medicaid/Medicare health insurance plans [9]. BeneFIT is a quasi-experimental, naturalistic study that compares two different implementation strategies developed by the health plans, without any usual care comparison group. The investigators serve as consultants to the health plans for both program design and delivery and are conducting a formal program evaluation. The health plans are responsible for conducting their programs, and both contracted with vendors to implement the mailings. The program is measuring implementation using mixed methods: quantitative (e.g., proportion of age-eligible enrollees mailed a FIT and delivered reminders, supporting activities by clinics, program costs) and qualitative interviews of key stakeholders to assess motivations, barriers, and facilitators of the program. Effectiveness outcomes are limited to what is easily measurable (e.g., FIT and colonoscopy completion rates using claims data). The Hybrid 3 study has no usual care comparison, as we knew already from the Hybrid 2 study that no mailings led to lower FIT completion rates.

### Program design and program delivery

Program design and delivery, the two new domains proposed for the hybrid studies, were distinctly different between the three studies. In Hybrid 1, the research team designed and built the EHR-linked mailed fecal testing program, trained clinical staff, and paid for their time, which helped to ensure fidelity to and completion of the intervention components. In Hybrid 2, the research team collaborated with the EHR vendor and health centers to design and build the EHR-embedded mailed FIT program, but the program itself was delivered by the clinic staff, with the research team assisting only with training and quality improvement activities. The timing, dose, and components of the intervention varied across health centers. For example, some health centers mailed letters and kits only once during the intervention year, while others completed monthly mailings. Some health centers combined the introductory letter mailing with the kit mailing and some required that the completed FIT be returned to the clinic, both were associated with decreased program effectiveness. The research provided clinics with small incentives but did not pay for program implementation. In Hybrid 3, the research team serves as consultants to the health plans and assists with evaluation but provides no training or implementation assistance. Hybrid 3 was paid for entirely by the health plans. They received an incentive for participation, but this was mainly to offset costs related to research, data capture, qualitative assessments, and time spent in meetings with the research staff.

## Discussion

The US Preventive Services Task Force recommends CRC screening for adults aged 50–75 because of strong evidence that it decreases CRC incidence and mortality [20]. However, screening rates remain suboptimal, particularly among disadvantaged populations [21]. The three studies we present were designed sequentially (informed by each prior study) to move evidence-based, research-tested interventions to increase CRC screening in diverse settings. The Hybrid 1 study, SOS, had the main goal of testing intervention effectiveness. It was embedded in a health care system, used automated data to identify eligible participants, used an EHR-linked registry to generate mailings, and employed clinic staff that worked in the delivery system to outreach to consented patients. Cost-effectiveness assessments were designed to inform decision-making about the spread of the program, and mixed methods explored potential refinements of the program and feasibility of implementation. The Hybrid 2 study, STOP CRC, moved the intervention into a community health center setting with a more diverse population. Key differences from the Hybrid 1 study were (1) inclusion of almost all age-eligible people, as consent was not required; (2) the collaborative process undertaken to design the program, which involved the EHR vendor, health center staff, and researchers; and (3) the implementation of the program solely by health center staff. The research team gave equal attention to evaluating overall program effectiveness and implementation by the community health centers. The Hybrid 3 study, BeneFIT, compares two different implementation strategies developed by the health plans, and was designed in part to decrease the implementation burden experienced in the Hybrid 2 study, STOP CRC. Each study answers important effectiveness and implementation questions and generated new questions.

We encountered some difficulties in trying to fit our 3 studies neatly into the hybrid framework. The Hybrid 1 study, SOS, is described as testing the effectiveness of an intervention on increasing CRC screening. However, one might argue that SOS instead tested different implementation strategies (i.e., the stepped interventions were implementation approaches). SOS had both “proof of concept” and pragmatic features. It was among the first trials to use EHR data to identify people and generate interventions for individuals’ overdue for CRC screening. It was also pragmatic in that it was embedded in a health care system and relied on employed clinic staff. As the primary outcome of the SOS trial was effectiveness of the intervention compared to usual care, we consider it to be an effectiveness trial, embedded in a real-world setting, but others might consider it to be an implementation study comparing different implementation methods, or an effectiveness trial only. Others have acknowledged that the distinction between effectiveness and implementation trials can be blurred [22].

Curran et al.’s framework domains omitted some distinguishing features we observed in our Hybrid 1, Hybrid 2, and Hybrid 3 studies, such as the role of research staff versus clinic/health plan staff in designing (or adapting) and implementing the intervention [3]. We think these features might be generalizable to other hybrid studies that progress along the research to practice continuum. Based on our three studies, we propose additional categories to the current hybrid typology.

### Program design

Collaboration with the target setting is often a critical component of implementation studies and helps to ensure a smoother implementation process. In the Hybrid 1 study, the researchers designed program elements and dose, while considering the resources available in that specific setting. In the Hybrid 2 study, researchers and healthcare centers collaboratively adapted the Hybrid 1 study components based on the new settings’ resources. In the Hybrid 3 study, the health plans implementing the program adapted the program components to fit their resources, with the researchers serving as consultants only.

### Program delivery

How an intervention is delivered is also a critical aspect of its transition from research to clinical practice. In our studies, the “agents” who delivered the intervention, as defined by Proctor et al. [5], shifted from tight to almost no research control. In the Hybrid 1 study, the research team delivered the mailings, and they also trained and monitored the clinical staff (nurses who made the follow-up calls). In the Hybrid 2 study, the research team assisted with training and implementation supports (PDSA cycles). In the Hybrid 3 study, the research team provided consultation services only, with the health plans delivering the program on their own, as would be the case once the program was institutionalized.

Our studies demonstrate that research-tested interventions for increasing CRC screening are rarely ready “right out of the box” for broad dissemination and may explain in part the long transition from research to practice. Even when the intervention is found to be effective, cost-effective, feasible, and acceptable, transitioning the intervention to day-to-day practice is a stepped and nuanced process, and one size may not fit all. Furthermore, contextual factors may lead to diminution of intervention effectiveness, resulting in additional research questions and further program adaptations.

Our analysis of the Hybrid 1, 2, and 3 SOS, STOP CRC, and BeneFIT studies has limitations. We describe the hybrid continuum of research to practice studies using only one area of research, CRC screening, and this continuum may be different than in other areas of health services implementation research. We also note that others may disagree with the hybrid category we assigned each study.

Our studies began prior to the hybrid design framework publication, and while our knowledge of the model may have influenced the design of our Hybrid Type 2 and 3 studies, we did not plan our progression a priori based on the framework. The progression of the three studies over a ten-year period was more organic, with each study answering questions posed by the prior study, and implementation progressively becoming more important than effectiveness, as the body of evidence on effectiveness grew. Lastly, the hybrid framework was intended to speed research into practice, which our studies may or may not demonstrate. Mailed FIT programs are burgeoning, but implementing sites face many implementation challenges and potential solutions that we and others continue to explore.

## Conclusion

Research-tested interventions are rarely ready for widespread dissemination. The hybrid models provide a framework for a series of progressive studies that test not just effectiveness, but also implementation, with each step progressing towards real-world practice. We present three studies that illustrate this process. We also found that the study continuum from an effectiveness focus to a focus on implementation may be nuanced and difficult to categorize clearly. We propose adding two new features that might be useful in characterizing hybrid studies: program design and program delivery, with a continuum from researcher-led in Hybrid 1 studies; researcher-organizational collaboration in Hybrid 2 studies; to organization-led in Hybrid 3 studies. Our three hybrid studies’ design and delivery became increasingly pragmatic, adaptable, and informed by the contexts, individuals, and organizations delivering them. Additional comparisons of Hybrid 1, 2, and 3 may help to confirm whether these new features are generalizable markers of studies along the pipeline from research to practice and whether they speed the uptake of research into practice.

## Abbreviations

BeneFIT: BeneFIT to increase Colorectal Screening in Priority Populations
CFIR: Consolidated framework for implementation research
CRC: Colorectal cancer
EHR: Electronic health record
FIT: Fecal immunochemical test
PDSA: Plan-Do-See-Act
RE-AIM: Reach, Effectiveness, Adoption, Implementation, Maintenance
SOS: Systems of Support to Increase Colorectal Cancer Screening and Follow-up
STOP CRC: Strategies and Opportunities to STOP Colon Cancer in Priority Populations
